# The group selection–inclusive fitness equivalence claim: not true and not relevant

**DOI:** 10.1017/ehs.2020.9

**Published:** 2020-04-23

**Authors:** Matthijs van Veelen

**Affiliations:** CREED, Universiteit van Amsterdam, Roetersstraat 11, 1018 WB Amsterdam, the Netherlands

## Abstract

The debate on (cultural) group selection regularly suffers from an inclusive fitness overdose. The classical view is that all group selection is kin selection, and that Hamilton's rule works for all models. I claim that not all group selection is kin selection, and that Hamilton's rule does not always get the direction of selection right. More importantly, I will argue that the paper by Smith (2020; Cultural group selection and human cooperation: a conceptual and empirical review. *Evolutionary Human Sciences*, **2**) shows that inclusive fitness is not particularly relevant for much of the empirical evidence relating to the question whether or not cultural group selection shaped human behaviour.

## Graphical abstract

**Figure:**
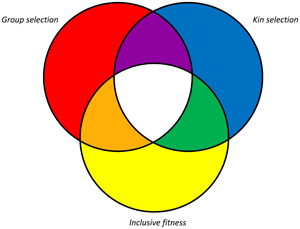

**Social media summary:** The group selection debate really benefits from moving past the ‘equivalence with inclusive fitness’ claim (which also happens to be false).

There is a long history of disagreement on group selection. As described also in the paper by Smith (2020), the first wave of dismissing group selection was inspired by Hamilton's rule. This rule suggests that selection takes place at the individual level, or maybe the gene level, but not at the group level (Hamilton 1964; Williams 1966).

Also later, when group selection, or multilevel selection, made a bit of a comeback, the arguments against them regularly came in combination with the claim that group selection and inclusive fitness are equivalent. If other group members would be sufficiently related, being altruistic towards them would be selected for. That would be kin selection, which we understand with Hamilton's rule (Queller 1992; Lehmann et al. 2007; see also Traulsen and Nowak 2006; and van Veelen et al. 2012).

Since the opponents of group selection generally care a lot about inclusive fitness, it seems that the proponents of group selection have chosen to pick their battles. They tend not to dispute the group selection–inclusive fitness equivalence, but accept that as a fact, and argue that equivalence does not mean that group selection does not exist, just that you can also understand it using Hamilton's rule (Sober and Wilson 1998).

For me as a group selection agnostic, but a math believer, this leaves me with two sides of a debate agreeing on something that I disagree with. For some group selection models (linear ones, in which how much of a difference it makes whether or not I cooperate is independent of how many other individuals in my group do) Hamilton's rule will indeed always get the direction of selection right (van Veelen 2011). For other ones, for instance if me cooperating only makes a difference if sufficiently many others do too, there is no meaningful definition of costs and benefits that make Hamilton's rule always point in the right direction. What is there, is a generally applicable way to reverse engineer the costs and benefits, so that they make Hamilton's rule hold (Nowak et al. 2017; van Veelen et al. 2017; van Veelen 2018).

The paper by Smith (2020) is not about group selection in general, but about cultural group selection. It is a heroic effort to gather all the empirical evidence in order to reach a verdict on whether or not cultural group selection has shaped human sociality. Now I am not an expert on cultural group selection, so I will leave the balancing of the evidence for and against to the other commenters, who actually know about this. What I will do, is express my appreciation of the fact that the author moves away from the standard approach to group selection dismissal, which is to suffocate all arguments with inclusive fitness. For many of the relevant empirical questions (about the nature of learning and imitation, how homogeneous groups are, or what norms do to behaviour), it turns out that inclusive fitness is simply irrelevant.

Also not good for the debate in the past is that kin selection and inclusive fitness got tangled up. Kin selection is a selection process by which certain behaviours towards kin can get selected for. It always involves some deviation from random matching. Hamilton's rule is a rule that may or may not get the direction of selection right. These are two different things. It is possible that Hamilton's rule gets the direction of selection wrong for a kin selection process (when fitness effects are not linear, but assortment is caused by identity by descent). It is also possible that it gets the direction of selection right for a selection process that is not kin selection (when fitness effects are linear, but assortment is not caused by identity by descent). Figure 1 depicts that.

**Figure 1.**
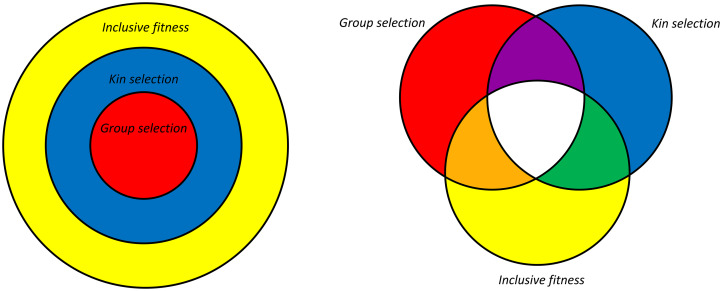
On the left is the classical view; all group selection models are kin selection models, and Hamilton's rule works for all models. On the right is the alternative, where all combinations are possible. For instance, group selection models where assortment is not based on identity by descent are not kin selection models. For models where fitness effects are not linear, Hamilton's rule, with meaningful definitions of costs and benefits, does not always get the direction of selection right.

In this respect, it is interesting to think whether or not the label kin selection could apply to cultural group selection. One perfectly legitimate choice would be to say no, because the word ‘kin’ should be reserved for genetic relatedness. Another legitimate choice would be to say that ideas are also transmitted, and therefore they can be shared because both individuals inherited them from the same individual. If ideas are shared because of identity by descent, then one could see the similarity with genetic kin. When people with certain norms or ideas preferentially interact with each other, then they are not similar because they are identical by descent, and therefore that would not be analogous to kin selection.

Finally, it is worth pointing out that the Price equation, which is also used in Smith (2020), is generally accepted as a tool in the (cultural) group selection literature, both by proponents and by opponents (Price 1970, 1972). That is unfortunate, because it inspires people to believe things to be true that are not. What this approach does, is borrow concepts from statistics (like regression coefficients) and apply those, not to statistical estimation, but to modelling. In this modelling context, they are used for doing things that statisticians specifically try to avoid. In statistics, regression coefficients are used to estimate parameters for a given model by averaging away the noise in the observations. For doing that properly, it is of crucial importance to get the model specification right, because if the model is misspecified, these regression coefficients become meaningless. The Price equation does not use regression coefficients for parameter estimation, but for trying to straightjacket possibly non-linear models into linear ones. In statistical terms, the Price equation is used to actively allow for misspecification, and for treating all models as if they are linear, even if they are not. This is not OK. For a detailed discussion of how the Price equation does everything that statisticians have forbidden, see van Veelen (2020). This paper also shows how using the Price equation helps obscure effects that are relevant to group selection, such as the cancellation effect at the group level (Akdeniz and van Veelen 2019).

## Research transparency and reproducibility

This commentary contains no data, materials, protocols or software.
